# Plasma exosomal lncRNA-related signatures define molecular subtypes and predict survival and treatment response in hepatocellular carcinoma

**DOI:** 10.3389/fimmu.2025.1663943

**Published:** 2025-10-15

**Authors:** Fangmin Zhong, Fangyi Yao, Xin-Lu Wang, Zihao Wang, Bo Huang, Jing Liu, Xiaozhong Wang, Lei Zhang

**Affiliations:** Jiangxi Province Key Laboratory of Immunology and Inflammation, Jiangxi Provincial Clinical Research Center for Laboratory Medicine, Department of Clinical Laboratory, The Second Affiliated Hospital, Jiangxi Medical College, Nanchang University, Nanchang, Jiangxi, China

**Keywords:** hepatocellular carcinoma, exosomal lncRNA, molecular subtype, prognosis, treatment response

## Abstract

**Background:**

Hepatocellular carcinoma (HCC) faces challenges in early diagnosis, prognosis, and treatment stratification due to molecular heterogeneity. This study aimed to establish a plasma exosomal long non-coding RNA (lncRNA)-based framework for molecular classification, prognostication, and therapeutic guidance in HCC.

**Methods:**

The transcriptomic data from 230 plasma exosomes and 831 HCC tissues were integrated. A competitive endogenous RNA (ceRNA) network was constructed via the miRcode, miRTarBase, TargetScan, and miRDB databases to define exosome-related genes (ERGs). Unsupervised consensus clustering was used to stratify HCC patients on the basis of ERG profiles. Prognostic models were developed and optimized via 10 machine learning algorithms with 10-fold cross-validation. Treatment responses were predicted via the SubMap, TIDE, and oncoPredict algorithms. RT-qPCR experiments were conducted to validate the expression of model genes.

**Results:**

We identified 22 dysregulated plasma exosomal lncRNAs in HCC. The upregulated lncRNAs formed a ceRNA network regulating 61 ERGs and were significantly enriched in cell cycle regulation, TGF-β signaling, the p53 pathway, and ferroptosis. ERG expression stratified HCC into three subtypes (C1–C3). The C3 subtype exhibited the poorest overall survival, advanced grade and stage, an immunosuppressive microenvironment (increased Treg infiltration, elevated PD-L1/CTLA4 expression, highest TIDE score), and hyperactivation of proliferation (MYC, E2F targets) and metabolic pathways (glycolysis, mTORC1). A random survival forest-derived 6-gene risk score (G6PD, KIF20A, NDRG1, ADH1C, RECQL4, MCM4) demonstrated high prognostic accuracy. High-risk patients presented increased TP53/TTN mutations and increased tumor mutational burdens. Risk model analysis predicted differential treatment responses: low-risk patients exhibited superior anti-PD-1 immunotherapy responses, whereas high-risk patients showed increased sensitivity to DNA-damaging agents (e.g., the Wee1 inhibitor MK-1775) and sorafenib. Experimental validation confirmed consistent dysregulation of the six-gene signature (G6PD, KIF20A, NDRG1, ADH1C, RECQL4, MCM4) in HCC cell lines, reinforcing the model’s biological relevance.

**Conclusion:**

Plasma exosomal lncRNAs enable robust molecular subtyping, accurate prognostic stratification, and treatment response prediction in HCC. The ERG-centric classification system and validated 6-gene risk model provide clinically actionable tools for precision oncology.

## Introduction

Hepatocellular carcinoma (HCC) represents a global health crisis, accounting for 90% of primary liver cancers and causing over 830,000 annual deaths worldwide (1, 2). Despite advances in targeted therapies and immunotherapy, the five-year survival rate for advanced HCC patients remains below 20%, largely because of late diagnosis and heterogeneous treatment responses (3). Current diagnostic biomarkers such as alpha-fetoprotein (AFP) exhibit limited sensitivity for early-stage detection (4), whereas conventional imaging fails to identify micrometastatic disease (5). These clinical challenges underscore the urgent need for novel molecular stratification tools.

Tumor-derived exosomes—nanoscale vesicles (30–150 nm) carrying bioactive molecules—have emerged as pivotal mediators of hepatocarcinogenesis (6). Among their cargo, long non-coding RNAs (lncRNAs) function as competitive endogenous RNAs (ceRNAs), sequestering microRNAs to derepress oncogenic transcripts (7). For example, exosomal H19 drives metastasis through miR-520a-3p/LIMK1 axis activation (8), whereas HEIH induces immunosuppression via STAT3 upregulation (9). These molecules offer unique advantages as liquid biopsy biomarkers because of their stability in circulation and tumor-specific expression patterns.

Critical knowledge gaps persist in translating exosomal lncRNA biology to clinical practice. Comprehensive characterization of plasma exosomal lncRNA profiles in large HCC cohorts remains limited, and the functional architecture of their ceRNA networks is poorly mapped. Moreover, the prognostic utility of exosome-derived signatures for molecular subtyping and therapy prediction is virtually unexplored, particularly compared with that of tissue-based approaches.

To address these unmet needs, we integrated transcriptomic data from 230 plasma exosome samples (112 HCC patients vs 118 healthy controls) through a multiomics framework. This study aimed to identify dysregulated exosomal lncRNAs, construct ceRNA networks, define exosome-related genes (ERGs), elucidate their molecular landscapes, establish ERG-driven molecular subtypes, and develop a machine learning-based prognostic model for personalized risk stratification and therapeutic guidance. Additionally, we experimentally validated the expression patterns of the prognostic signature genes to establish their clinical applicability.

## Materials and methods

### Data collection and preprocessing

This study integrated 831 hepatocellular carcinoma (HCC) samples from three major public databases, including The Cancer Genome Atlas (TCGA-LIHC, n=370, https://portal.gdc.cancer.gov/projects/TCGA), the Gene Expression Omnibus (GSE14520, n=221, https://www.ncbi.nlm.nih.gov/geo/), and the International Cancer Genome Consortium (ICGC-LIRI, n=240, https://dcc.icgc.org/). The RNA-seq data from TCGA-LIHC and ICGC-LIRI were downloaded as raw counts and uniformly transformed to Transcripts Per Million (TPM) values, followed by log2 transformation. The microarray data from GSE14520 were used as provided by the authors after log2 transformation and quantile normalization. The exosomal lncRNA expression matrix from exoRBase 2.0 database (10) was also log2(TPM+1) transformed, covering plasma exosomal transcriptome data from 112 HCC patients and 118 healthy individuals. Somatic mutation and copy number variation data were obtained through the TCGA portal.

### Construction of the ceRNA regulatory network

We focused on the upregulated exosomal lncRNAs for ceRNA network construction because they are more likely to act as oncogenic ‘sponges’ that sequester miRNAs and release the inhibition on target mRNAs, thereby driving HCC progression. The use of three stringent miRNA-mRNA interaction databases (miRTarBase, TargetScan, miRDB) was to ensure the reliability and biological relevance of the predicted interactions, minimizing false positives. A multilevel strategy was adopted: first, miRNA binding sites of differentially expressed lncRNAs were predicted via the miRcode database (11); subsequently, the miRTarBase (12), TargetScan (13), and miRDB (14) databases were integrated, retaining the miRNA–mRNA relationships supported by all three databases; finally, the intersection of target genes of differentially expressed lncRNAs and upregulated mRNAs in HCC tissues (|logFC|>1, FDR<0.05) was used to define exosome–related genes (ERGs), and a ternary regulatory network was constructed via Cytoscape 3.9.1.

### Functional and immune analyses

The following steps were executed: GO/KEGG pathway enrichment analysis was conducted via the clusterProfiler package (FDR<0.05 significance threshold) (15), hallmark pathway activity was quantified via gene set variation analysis (GSVA), pathway differences between risk groups were compared via gene set enrichment analysis (GSEA), and the abundance of 22 immune cell types was analyzed via the CIBERSORT algorithm (LM22 signature matrix) (16).

### Identification of molecular subtypes

On the basis of the ERG expression profile, unsupervised consensus clustering was performed via the ConsensusClusterPlus package (17): the Pearson distance metric, PAM clustering algorithm, 80% resampling ratio, and 1000 iterations were adopted, and the optimal number of subtypes (k=3) was determined according to the cumulative distribution function (CDF) curve.

### Development of the prognostic model

The TCGA-LIHC cohort was utilized as the training set, while the remaining cohorts served as validation sets. Ten machine learning algorithms—CoxBoost, stepwise Cox, Lasso, Ridge, elastic net (Enet), survival support vector machines (survival-SVMs), generalized boosted regression models (GBMs), supervised principal components (SuperPC), partial least squares Cox (plsRcox), and random survival forest (RSF)—were systematically integrated under a 10-fold cross-validation framework, resulting in 118 distinct configurations. For the CoxBoost model, the optimal penalty (shrinkage) value was first identified using the “optimCoxBoostPenalty” function. This value was then combined with 10-fold cross-validation to determine the optimal number of boosting steps. The model was subsequently fitted with the “CoxBoost” function. Stepwise Cox regression was performed using the survival package, with model complexity evaluated based on the Akaike Information Criterion (AIC). All possible configurations of the direction parameter—”both”, “backward”, and “forward”—were examined. The Lasso, Ridge, and Enet models were implemented using the “cv.glmnet” function from the glmnet package. The regularization parameter λ was selected via 10-fold cross-validation, while the mixing parameter α was varied from 0 to 1 in increments of 0.1: α = 1 corresponds to Lasso, α = 0 to Ridge, and intermediate values to Enet. The survival-SVM model was constructed using the “survivalsvm” function from the survivalsvm package, which applies support vector machine methodology to survival data. The GBM model was fitted using the “gbm” function from the gbm package with 10-fold cross-validation. The SuperPC model, an extension of principal component analysis (PCA), was implemented using the superpc package, with cross-validation performed via the “superpc.cv” function over 10 folds. The plsRcox model was developed using the “cv.plsRcox” function from the plsRcox package. The RSF model was built using the “rfsrc” function from the randomForestSRC package, with two key parameters: “ntree” (the number of trees in the forest, set to 1000) and “nodesize” (the minimum size of terminal nodes, set to 5). Within a 10-fold cross-validation framework, the concordance index (C-index) was used as the evaluation metric to optimize hyperparameters, and a risk score model based on 6 key genes was ultimately constructed. The model was trained on the TCGA training set and independently tested on the ICGC/GSE14520 validation set.

### Prediction of treatment response

A dual-dimensional strategy was employed: drug sensitivity was calculated on the basis of the GDSC2 database via oncoPredict to determine IC_50_ values (18); immune therapy response was evaluated via SubMap analysis (GenePattern platform) to assess the transcriptional similarity between high- and low-risk groups and samples treated with anti-PD-1/CTLA-4 (Bonferroni-corrected p<0.05); and the immune escape potential was quantified via the TIDE algorithm (http://tide.dfci.harvard.edu).

### Cell culture

Human hepatocellular carcinoma cell lines (HLE, Huh7, and Hep3B) and immortalized normal hepatocyte cell lines (WRL68 and THLE-3) were obtained from the American Type Culture Collection (ATCC) or the National Collection of Authenticated Cell Cultures (China). All cells were cultured in Dulbecco’s Modified Eagle Medium (DMEM) or RPMI-1640 medium supplemented with 10% fetal bovine serum (FBS) and 1% penicillin/streptomycin (all from Gibco, USA). Cells were maintained at 37°C in a humidified incubator with 5% CO_2_.

### Quantitative real-time polymerase chain reaction

Total RNA was extracted from different HCC cell lines using Trizol reagent (Takara, Japan). The RNA was then reverse transcribed into complementary DNA (cDNA) using the PrimeScript RT reagent kit (Takara, Japan) according to the manufacturer’s instructions. Gene expression analysis was performed by RT-qPCR using TB Green Premix Ex Taq (Takara, Japan) on an ABI7500 instrument, with primers listed in **Supplementary Table S1**. The internal control GAPDH was utilized and the 2^-ΔΔCt^ values were normalized to the levels of GAPDH for data analysis.

### Statistical analysis

The analyses were performed in the R 4.2.1 environment: differences between continuous variable groups were evaluated via the Wilcoxon rank sum test (for two groups) or the Kruskal–Wallis test (for multiple groups); categorical variables were analyzed via Fisher’s exact test; survival analysis was conducted via Kaplan–Meier curves and the log-rank test; prognostic factors were assessed via univariate and multivariate Cox regression; correlations were calculated via the Spearman rank correlation coefficient; the significance threshold was set at p < 0.05 (two-tailed test); and multiple testing correction was performed via the Benjamini–Hochberg method.

## Results

### Molecular characteristics and potential regulatory mechanisms of lncRNAs in HCC exosomes

**Figure 1:**
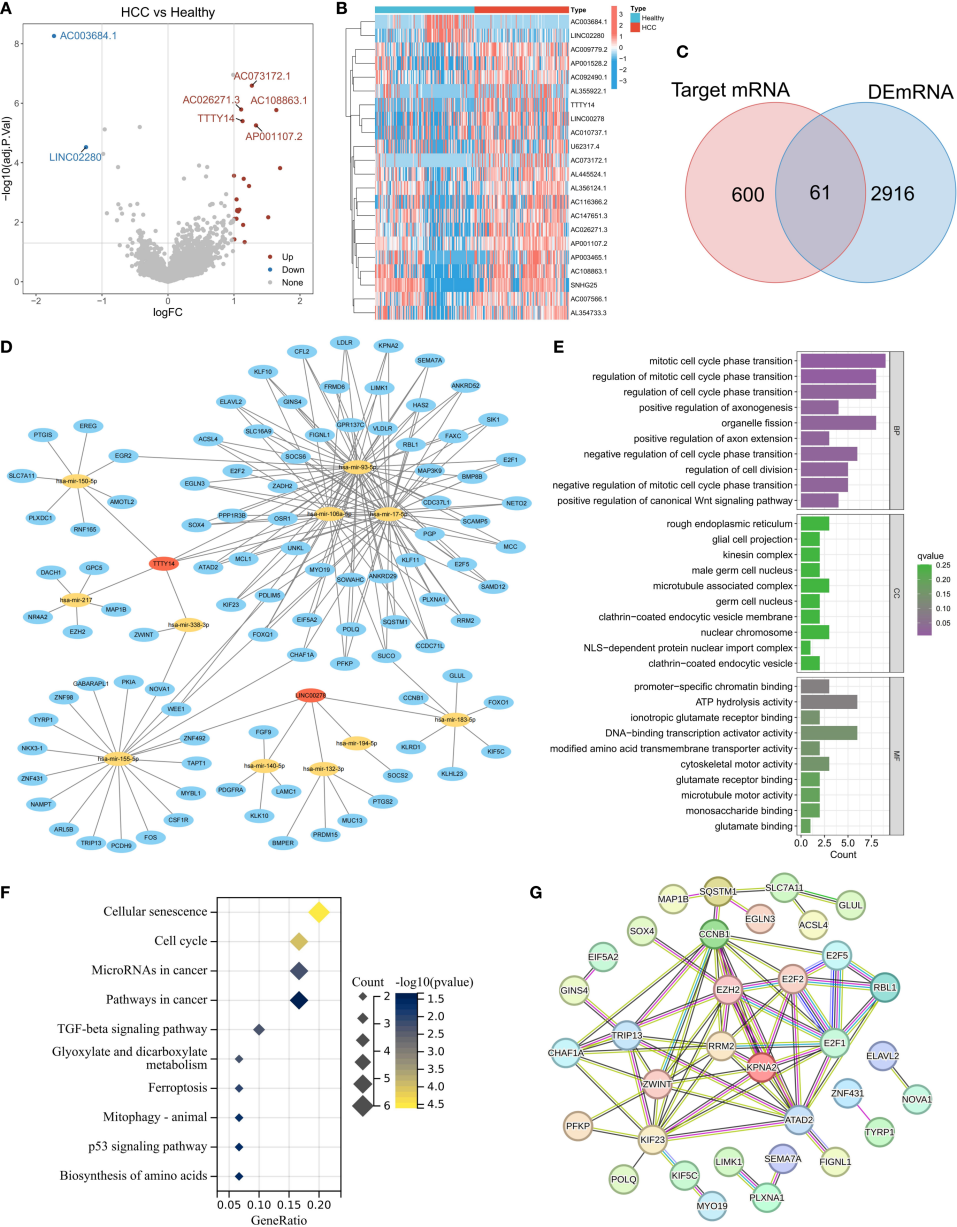
Expression characteristics of lncRNAs derived from exosomes in the peripheral blood of patients with HCC. **(A)** Heatmap of lncRNA expression in exosomes from the HCC and normal groups. **(B)** Volcano plot of differentially expressed lncRNAs (screening criteria: |logFC|>1, adj.p<0.05). **(C)** Intersection of target genes of differentially expressed lncRNAs and upregulated mRNAs in HCC (defined as ERGs). **(D)** lncRNA–miRNA-ERG ceRNA regulatory network. **(E-G)** Functional annotation of ERGs: GO enrichment analysis **(E)**, KEGG pathway enrichment **(F)**, and PPI network core nodes **(G)**.

Using the exoRBase database, we analyzed the RNA expression profiles of blood-derived exosomes from 118 healthy individuals and 112 HCC patients. Differential expression analysis (selection criteria: |logFC| > 1, adjusted P value < 0.05) identified 22 lncRNAs that were differentially expressed between the two groups (**Figure 1A**). Among them, AC003684.1 and LINC02280 were significantly downregulated in the HCC samples, whereas 20 lncRNAs, such as AC073172.1, AC026271.3, AC108863.1, TTTY14, and AP001107.2, were significantly upregulated (**Figure 1B**). To reveal the potential regulatory mechanisms of exosomal lncRNAs, we focused on upregulated lncRNAs and constructed a related ceRNA network. The specific process included predicting miRNAs bound to lncRNAs via the miRcode database; predicting the target genes of these miRNAs via the miRDB, miRTarBase, and TargetScan databases (**Figure 1C**); and finally, taking the intersection of these target genes and the upregulated mRNAs in HCC samples, a total of 61 genes were obtained and defined as exosome-related genes (ERGs) (**Figure 1D**). The main functions of ERG include mitotic cell cycle phase transition, organelle fission, promoter-specific chromatin binding, and DNA-binding transcription activator activity. KEGG pathway enrichment analysis revealed that ERG was significantly enriched in cellular senescence, the cell cycle, microRNAs in cancer, and cancer pathways, involving key pathways such as the TGF-beta and P53 signaling pathways, as well as metabolism, ferroptosis, and mitochondrial autophagy-related pathways. These results suggest that ERG is closely related to the proliferation and growth of cancer cells. Protein–protein interaction (PPI) network analysis further identified core hub molecules such as RRM2, ZWINT, KPNA2, EZH2, E2F1, E2F2, TRIP13, KIF23, and CCNB1.

### Analysis of the molecular landscape of ERGs and related tumor clustering subtypes

**Figure 2:**
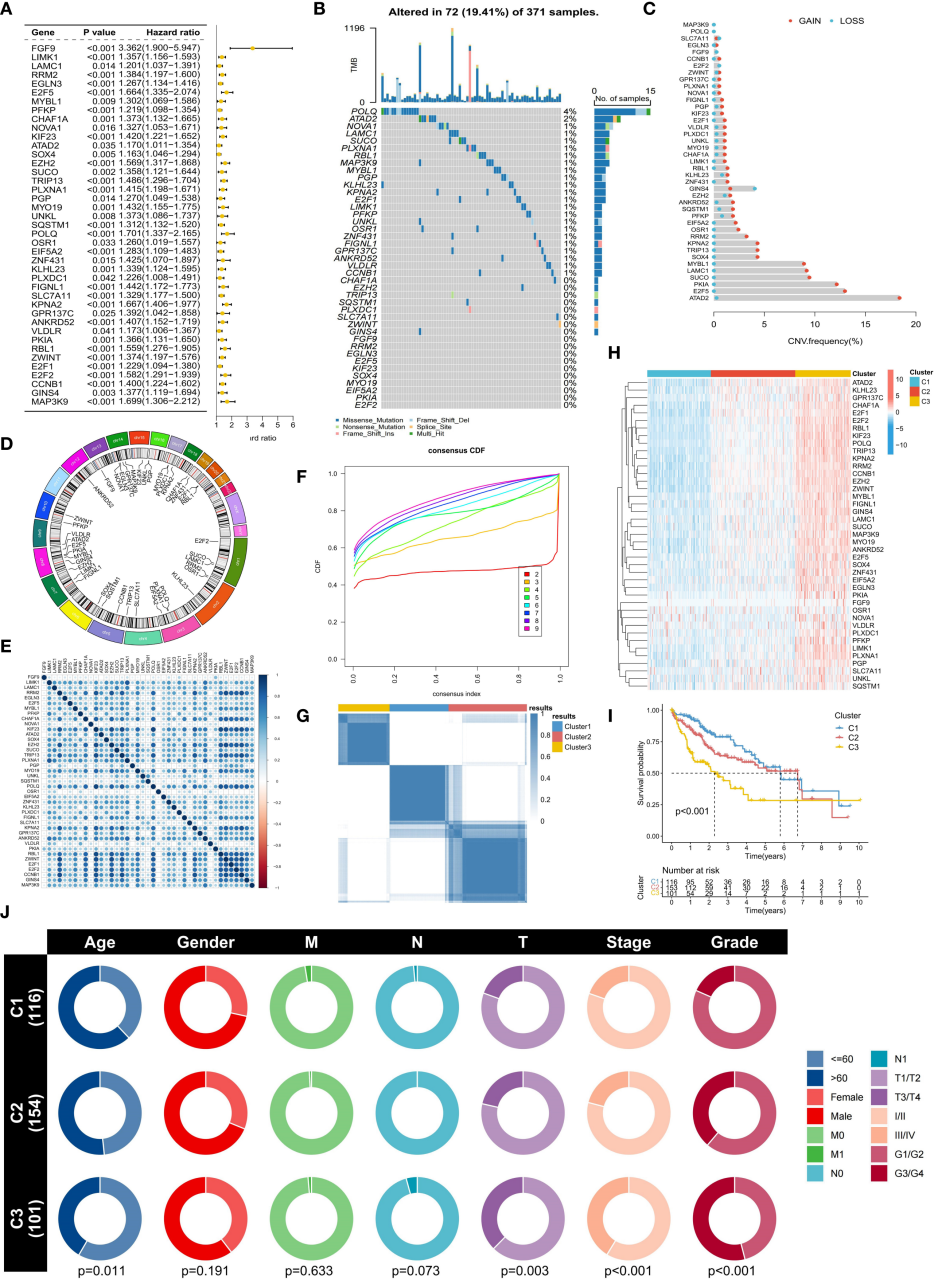
Molecular characteristics and subtype analysis of ERGs in HCC. **(A)** Univariate Cox regression survival analysis of ERGs (p<0.05). **(B)** Somatic mutation spectrum of ERG in HCC samples. **(C)** Frequency of ERG copy number variation (CNV). **(D)** Chromosomal location distribution of ERGs. **(E)** Correlation analysis of ERG expression. **(F)** Cumulative distribution function (CDF) distribution under different numbers of clustering clusters. **(G)** Consensus clustering based on ERG expression (divided into C1-C3 subtypes). **(H)** Comparison of ERG expression levels among subtypes. **(I)** Differences in overall survival among subtypes. **(J)** Association analysis of subtypes with clinicopathological features.

To further explore the molecular characteristics of ERG, we conducted a univariate Cox regression analysis and found that 41 ERGs were significantly associated with the prognosis of HCC patients (p < 0.05) (**Figure 2A**). The hazard ratios (HRs) of these genes were all greater than 1, indicating that they are poor prognostic factors. Somatic mutation analysis revealed that 19.41% of the HCC samples had ERG gene mutations, but the mutation frequency of individual genes was relatively low (e.g., POLQ: 4%, ATAD2: 2%) (**Figure 2B**). Copy number variation (CNV) analysis revealed that most ERGs (such as ATAD2, E2F5, PKIA, SUCO, LAMC1, and MYBL1) presented significant copy number amplification in HCC samples, which might be related to their upregulated expression (**Figure 2C**). **Figure 2D** shows the distribution of ERGs on chromosomes, which were mainly concentrated on chromosomes 7, 8, and 14. Expression correlation analysis revealed a significant positive correlation among ERGs, suggesting their potential synergistic role in HCC (**Figure 2E**). These results collectively indicate that the overall abnormal expression of ERGs is an important cause of the malignant progression of HCC. Based on the expression profiles of ERGs, we subsequently classified HCC patients using an unsupervised consensus clustering algorithm. We observed that the relative change in the area under the CDF curve was larger when the number of clusters was 3 compared to 2, and the CDF distribution was flatter (**Figure 2F**). Moreover, as the number of clusters increased, the area under the CDF curve changed little, indicating that further increasing the number of clusters would not significantly enhance clustering stability. Therefore, we divided the HCC patients into three distinct subtypes (Clusters C1, C2, and C3) (**Figure 2G**). The overall expression level of ERGs was the lowest in the C1 subtype, followed by C2, and the highest in C3 (**Figure 2H**). Survival analysis confirmed that patients in the C1 subtype had the best prognosis, followed by those in the C2 subtype, and those in the C3 subtype had the worst prognosis, further validating the association between high ERG expression and poor prognosis (**Figure 2I**). Clinical feature correlation analysis revealed significant differences in T stage, clinical stage, and histological grade among the different clustering subtypes (**Figure 2J**). Specifically, the proportion of patients with advanced T stage, clinical stage, and high histological grade increased from C1 to C2 and then to C3, reflecting an increasing trend in tumor malignancy, indicating that our molecular classification accurately captures and reflects the continuum of HCC clinical aggressiveness.

### Biological characterization of ERG-related subtypes

**Figure 3:**
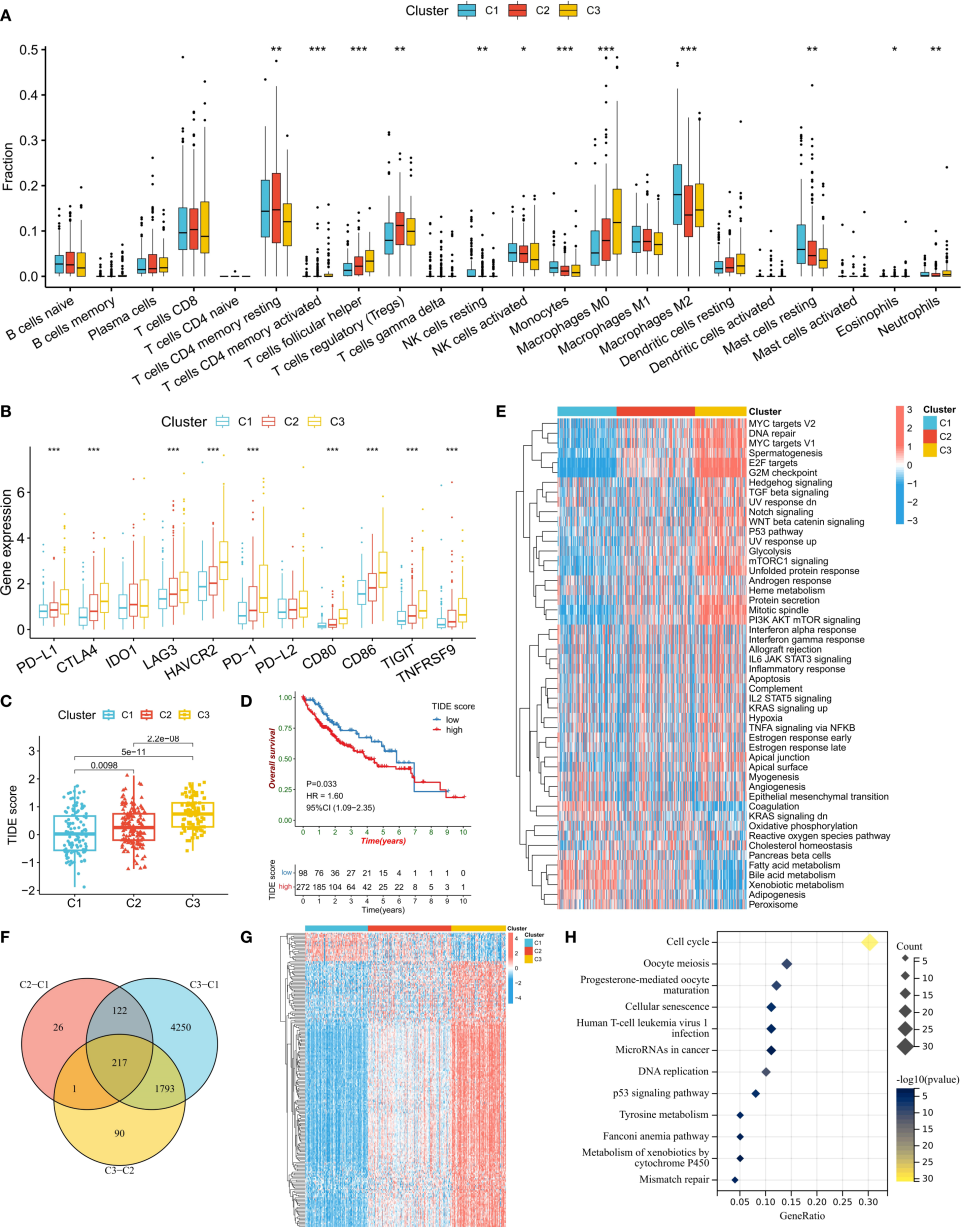
Biological characteristics of the ERG subtypes. **(A)** Differences in immune cell infiltration abundance among subtypes. **(B)** Comparison of immune checkpoint gene expression levels. **(C)** Distribution of TIDE scores among subtypes. **(D)** Correlations between TIDE scores and patient prognosis. **(E)** Heatmap of tumor marker pathway activity scores. **(F, G)** Screening and expression patterns of differentially expressed genes (DEGs) among subtypes. **(H)** KEGG pathway enrichment analysis of DEGs. *P<0.05, **P<0.01, ***P<0.001.

We further analyzed the differences in biological characteristics among the different subtypes. Immune cell infiltration analysis revealed that the C1 subtype was enriched in M2 macrophages, monocytes, resting NK cells, and resting mast cells, whereas the C2 and C3 subtypes were enriched in follicular helper T cells (Tfhs), regulatory T cells (Tregs), and M0 macrophages (**Figure 3A**). In addition, the proportion of activated NK cells was the lowest in the C3 subtype. The expression levels of immune checkpoint genes also significantly differed among the different subtypes, with PD-L1, CTLA4, LAG3, HAVCR2, PD-1, CD80, CD86, TIGIT, and TNFRSF9 showing stepwise increases from C1 to C2 and then to C3 (**Figure 3B**). A high tumor immune dysfunction and exclusion (TIDE) score indicates an increased possibility of tumor cell immune escape (**Figure 3C**). The analysis results revealed that the TIDE score was the lowest in the C1 subtype and the highest in the C3 subtype. Survival analysis also confirmed that a high TIDE score was significantly associated with a worse prognosis in HCC patients (**Figure 3D**). These results suggest that the increased infiltration of immunosuppressive cells (such as Tregs) and the upregulated expression of immune checkpoint genes in the C3 subtype might jointly induce the formation of an immunosuppressive tumor microenvironment (TME), leading to poor patient prognosis.

We also compared the activity levels of tumor hallmark signaling pathways among different subtypes. The results revealed that in the C3 subtype, the activity levels of cell proliferation-related pathways (such as MYC targets, DNA repair, E2F targets, and the G2M checkpoint), oncogenic pathways (such as TGF beta, Notch, WNT beta catenin, and P53), and metabolic pathways (such as glycolysis and mTORC1 signaling) were the highest, indicating that the cancer cells in this subtype were highly proliferative (**Figure 3E**). To confirm the biological pathway characteristics of the subtypes, we conducted a differential expression gene (DEG) analysis for the three subtypes (**Figure 3F**). The results revealed 217 genes that were differentially expressed among the subtypes, with the majority of DEGs having the lowest expression in the C1 subtype and the highest in the C3 subtype (**Figure 3G**). KEGG enrichment analysis of these DEGs revealed that the most enriched pathway was the cell cycle pathway, further confirming the rapid proliferation ability of C3 subtype cells (**Figure 3H**).

### Construction and validation of the ERG-related risk score prognostic model

**Figure 4:**
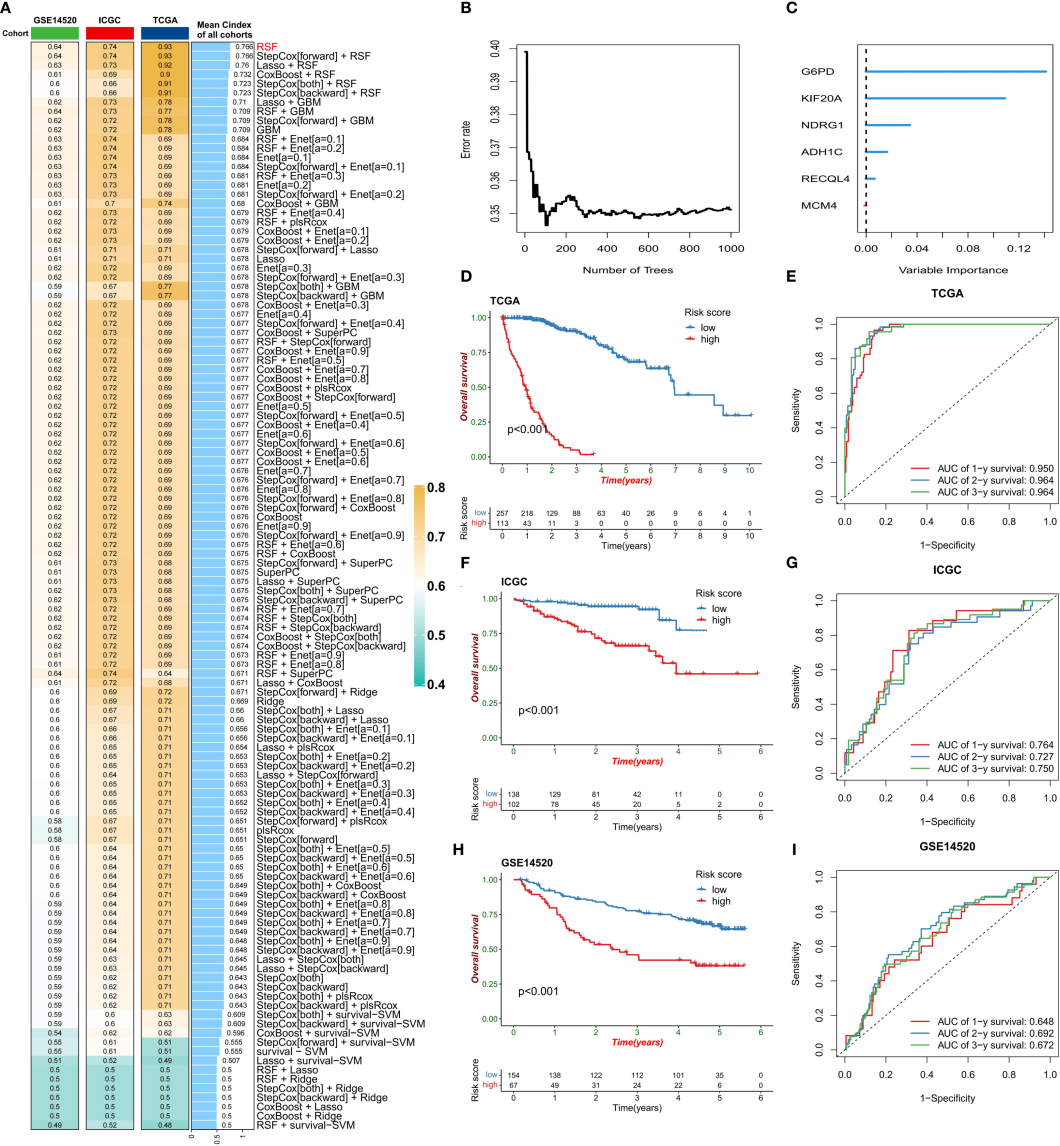
Construction of the risk score model. **(A)** Comparison of the prognostic prediction performance of 118 machine learning algorithms (sorted by the average C-index). **(B)** Relationship between the error rate of the random survival forest (RSF) model and the number of decision trees. **(C)** Ranking of gene importance in the final model. **(D-I)** Model validation: KM survival curves **(D, F, H)** and ROC curves **(E, G, I)** of the training set (TCGA) and validation set (ICGC/GSE14520).

**Figure 5:**
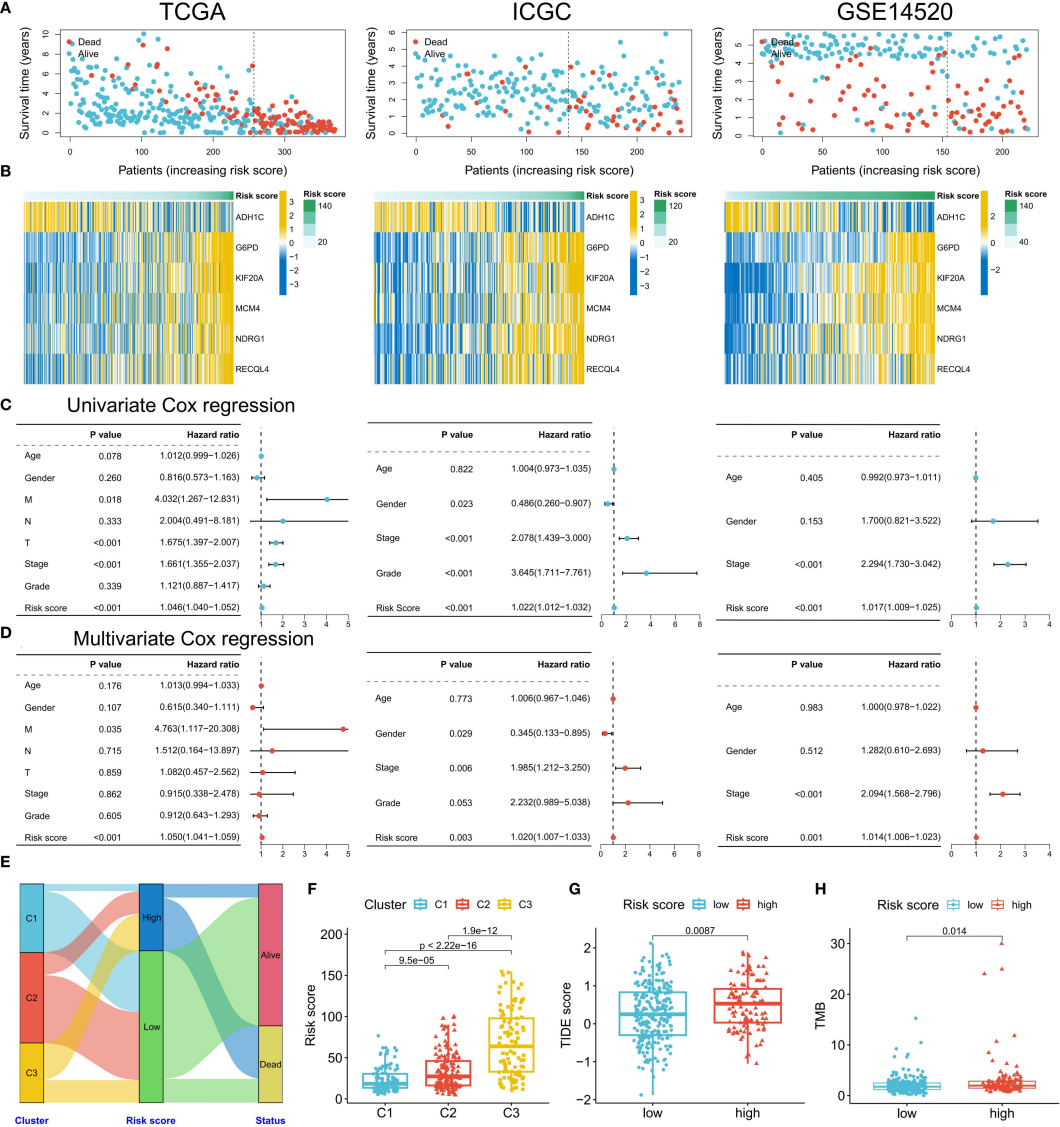
Clinical validation of the risk model. **(A)** Distribution of survival status in the high- and low-risk groups in the three cohorts. **(B)** Correlation between the risk score and model gene expression. **(C, D)** Univariate/multivariate Cox regression analysis of the risk score (TCGA/ICGC/GSE14520). **(E)** Association analysis of risk groups and clustering subtypes. **(F-H)** Comparison of risk scores among subtypes **(F)**, TIDE scores between the high- and low-risk groups **(G)**, and TMB levels **(H)**.

Given the strong association between ERG and HCC prognosis, we further explored its prognostic value. First, we constructed prognostic risk score models using 118 combinations of 10 machine learning algorithms (**Figure 4A**). Among them, the random survival forest (RSF) algorithm model had the highest average C-index in all cohorts, and the error rate was the lowest when the number of trees was 110 (**Figure 4B**). On the basis of this optimal model, we selected six key genes (G6PD, KIF20A, NDRG1, ADH1C, RECQL4, and MCM4) to construct the final risk score model and calculate the risk score for each sample (**Figure 4C**). Patients in the TCGA training cohort were divided into high-risk score and low-risk score groups on the basis of the optimal cutoff value. K–M curve analysis revealed that the prognosis of patients in the high-risk score group was significantly worse than that of patients in the low-risk score group (**Figure 4D**). The area under the receiver operating characteristic curve (AUC) values of this risk score model for predicting 1-year, 2-year, and 3-year overall survival (OS) in HCC patients were 0.950, 0.964, and 0.964, respectively, demonstrating extremely high prognostic predictive accuracy (**Figure 4E**). In two independent validation cohorts (the ICGC cohort and the GSE14520 cohort), the prognosis of patients in the high-risk score group was also significantly worse than that in the low-risk score group, further confirming the clinical predictive value of a high-risk score for poor prognosis. The ROC curve also verified the predictive efficacy of this risk score for OS: in the ICGC cohort, the AUC values for predicting 1-year, 2-year, and 3-year OS were 0.764, 0.727, and 0.750, respectively; in the GSE14520 cohort, they were 0.648, 0.692, and 0.672, respectively (**Figures 4F-I**). In the three HCC cohorts, the proportion of deceased patients in the high-risk score group was significantly greater than that in the low-risk score group (**Figure 5A**). Among the six model genes, the expression levels of G6PD, KIF20A, NDRG1, RECQL4, and MCM4 increased with increasing risk score, whereas the expression of ADH1C exhibited the opposite trend (**Figure 5B**). Both univariate and multivariate Cox regression analyses confirmed that this risk score model had significant independent prognostic predictive value in the three HCC cohorts (P < 0.01) (**Figures 5C, D**).

### Differences in pathological characteristics among patients in different risk score groups

**Figure 6:**
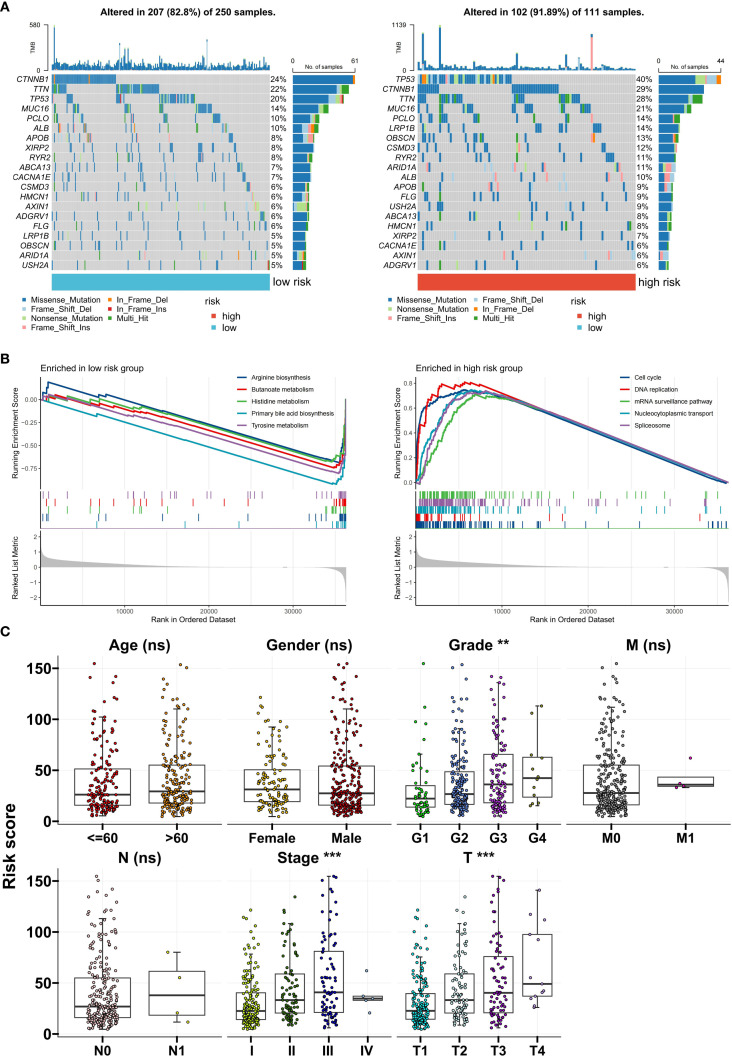
Clinical and genomic characteristics of risk groups. **(A)** Differences in somatic mutation spectra between the high- and low-risk groups. **(B)** Top 5 enriched pathways identified via GSEA. **(C)** Correlations between the risk score and clinical stage/grade. **P<0.01; ***P<0.001; ns, no significance.

Next, we analyzed the differences in clinical and genomic characteristics among patients in different risk score groups. In the TCGA cohort, we found that the low-risk score group was composed mainly of C1 and C2 subtype patients, whereas the high-risk score group included mainly C3 subtype patients (**Figure 5E**). The risk score was the lowest for the C1 subtype and the highest for the C3 subtype (**Figure 5F**). The high-risk score group also had higher TIDE scores and tumor mutation burdens (TMBs) (**Figures 5G, H**). Further analysis of somatic mutations revealed that the proportion of samples with gene mutations in the high-risk score group was greater than that in the low-risk score group (low-risk group: 82.8%; the high-risk group had a significantly greater mutation frequency of TP53, CTNNB1, and TTN than did the low-risk group (91.89%), suggesting that a higher gene mutation frequency may be associated with the poor prognosis of the high-risk group (**Figure 6A**). Gene set enrichment analysis (GSEA) revealed that the top 5 significantly enriched pathways in the high-risk group were the cell cycle, DNA replication, the mRNA surveillance pathway, nucleocytoplasmic transport, and spliceosome, all of which are related to cell proliferation, whereas the low-risk group was enriched mainly in metabolic pathways such as arginine biosynthesis, butanoate metabolism, histidine metabolism, primary bile acid biosynthesis, and tyrosine metabolism, indicating pathway heterogeneity (**Figure 6B**). In terms of clinicopathological features, the risk score gradually increased with increasing histological grade (grade), clinical stage (stage), and T stage (**Figure 6C**).

### Application of the risk score model in treatment decision-making

**Figure 7:**
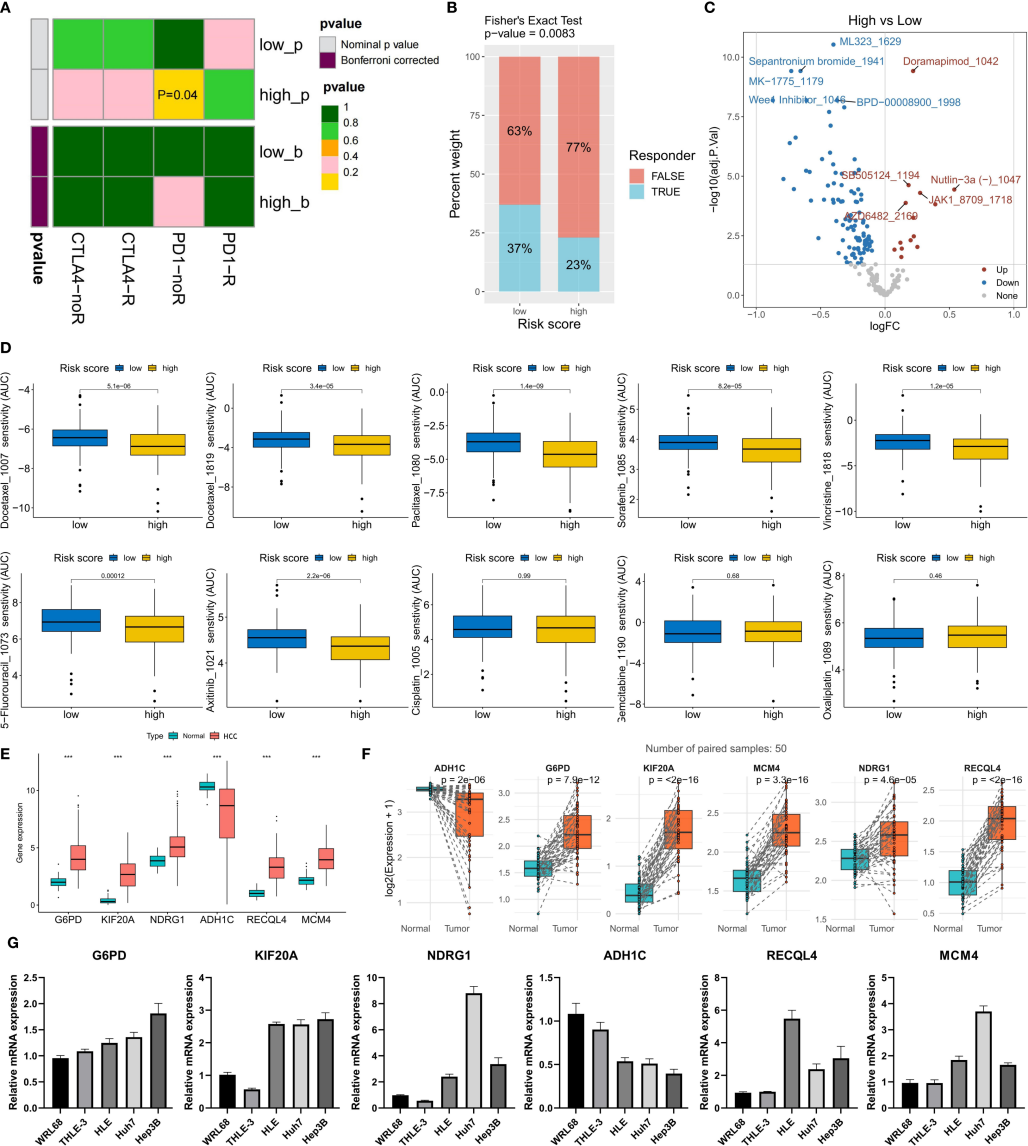
Risk model guiding treatment decisions and validation of model gene expression. **(A)** Prediction of the anti-CTLA-4/PD-1 immunotherapy response via the submap algorithm. **(B)** Prediction of the immunotherapy response ratio via the TIDE algorithm. **(C)** Top 5 drugs with differences in sensitivity between the high- and low-risk groups (IC50 comparison). **(D)** Prediction of sensitivity to 9 commonly used HCC chemotherapy drugs. **(E, F)** The expression differences of the six model genes between non-paired **(E)** and paired **(F)** samples of tumor and normal tissues in the TCGA-LIHC cohort. **(G)** The expression levels of the six model genes in two normal liver cell lines (WRL68 and THLE-3) and three HCC cell lines (HLE, Huh7 and Hep3B). ***P<0.001.

Finally, we evaluated the potential response of different risk score groups to treatment strategies. Given that the high-risk score group had a higher TIDE score, we used the submap algorithm to predict the response of this group to immune checkpoint inhibitors (ICIs). The results revealed that the high-risk score group had a lower response to anti-PD-1 immunotherapy (**Figure 7A**), whereas a greater proportion of patients in the low-risk score group were predicted to respond to immunotherapy (**Figure 7B**). With respect to chemotherapy and targeted drugs, we used the oncoPredict algorithm to predict the drugs with the greatest and smallest differences in drug sensitivity between the high- and low-risk score groups. Compared with the low-risk group, the high-risk group presented significantly lower half-maximal inhibitory concentration (IC50) values for ML323, doramapimod, sepantronium bromide, MK-1775, BPD-00008900, and Wee1 inhibitor, indicating greater treatment sensitivity (**Figure 7C**). Similarly, the low-risk score group was more sensitive to doramapimod, SB505124, Nutlin-3a (-), a JAK1 inhibitor, and AZD6482 (**Figure 7C**). We also explored the differences in the response of different risk score groups to 9 commonly used anticancer drugs for HCC. The prediction results revealed that patients in the high-risk score group were more sensitive to docetaxel, paclitaxel, sorafenib, vincristine, 5-fluorouracil, and axitinib, whereas there was no significant difference in sensitivity to cisplatin, gemcitabine, or oxaliplatin between the two groups (**Figure 7D**). These results provide potential treatment references for patients in different risk score groups and may help in the development of individualized treatment plans.

### Validation of the expression of risk scoring model genes

To explore the expression of the genes included in the risk scoring model, we compared the expression levels of these six genes in HCC tumor tissues and normal tissues from the TCGA-LIHC cohort. The analysis results showed that the expression levels of G6PD, KIF20A, NDRG1, RECQL4, and MCM4 were significantly higher in HCC tumor tissues than in normal tissues, while ADH1C was significantly downregulated in HCC tumor tissues (**Figure 7E**). In 50 pairs of HCC tumor tissues and matched normal tissues, we observed the same expression trends for these six genes (**Figure 7F**). Additionally, we evaluated the expression of these six genes in five cell lines: two normal liver cell lines (WRL68 and THLE-3) and three HCC cell lines (HLE, Huh7, and Hep3B). The results indicated that compared with normal liver cell lines, the expression of G6PD, KIF20A, NDRG1, RECQL4, and MCM4 was significantly upregulated, while ADH1C was significantly downregulated in HCC cell lines (**Figure 7G**). This result verified the expression characteristics of the six model genes in HCC samples.

## Discussion

HCC faces severe challenges due to delayed diagnosis and heterogeneous treatment responses (19). This study, by integrating RNA-seq data from 230 plasma exosome samples, established for the first time a molecular classification system and prognostic model based on exosomal lncRNAs, providing a new perspective for resolving the predicament of the precise diagnosis and treatment of HCC. Furthermore, the ERG-driven stratification of HCC into three subtypes (C1-C3) transcends mere prognostic separation; it unveils distinct biological entities with direct therapeutic implications.

The ceRNA regulatory network of exosomal lncRNAs is a significant finding of this study. The 22 differentially expressed lncRNAs [such as the oncogenes SNHG25 (20) and TTTY14 (21)] identified in the plasma of HCC patients competitively bind to miRNAs to regulate 61 ERGs. These genes dominate cell cycle progression (RRM2, CCNB1) (22, 23), DNA repair (E2F1) (24), and epigenetic remodeling (EZH2) (25) and are significantly enriched in the TGF-β, p53, and ferroptosis pathways. This finding confirms the role of exosome-derived lncRNAs and their related ceRNA networks in promoting HCC progression, as revealed by previous studies (7, 26).

Molecular classification on the basis of ERG expression profiles (C1-C3 subtypes) has clear clinical importance. The C3 subtype, which has high ERG expression, has the poorest prognosis and is accompanied by an advanced T stage and a high proportion of poorly differentiated tissues. Its immune microenvironment shows inhibitory characteristics: increased infiltration of regulatory T cells (Tregs), upregulated expression of immune checkpoint genes (PD-L1, CTLA4), and a significantly elevated TIDE score. Notably, C3 tumors simultaneously activate proliferation-related pathways (MYC, E2F targets) and metabolic reprogramming (mTORC1, glycolysis), and this synergistic effect may be a key driver of the invasive phenotype of these tumors. By distinguishing these populations based on plasma exosomal lncRNA signatures, our classification system provides a minimally invasive tool to guide first-line therapy selection and overcome the challenge of treatment heterogeneity in HCC.

The prognostic risk model constructed in this study has significant clinical value. The six ERG markers (G6PD, KIF20A, NDRG1, ADH1C, RECQL4, and MCM4) selected by the random survival forest (RSF) algorithm achieved an AUC of 0.964 for 3-year survival prediction in the training cohort and were successfully validated in the ICGC/GSE14520 cohort. Patients in the high-risk group have an increased frequency of TP53/TTN mutations and tumor mutational burden (27, 28), and the risk score is positively correlated with tissue grade and clinical stage. Crucially, we validated the expression of the six-gene risk signature in HCC clinical samples and cell lines. Consistent with bioinformatic predictions, G6PD, KIF20A, NDRG1, RECQL4, and MCM4 were significantly upregulated in HCC tissues and cell lines, whereas ADH1C was downregulated. This orthogonal validation confirms the robustness of our risk model and its foundation in HCC biology. The robust prognostic performance of our six-gene signature is further underpinned by the extensively documented, and often complementary, oncogenic roles of its constituents in HCC pathogenesis, as established in independent functional studies. This signature encapsulates genes that coordinately drive key hallmarks of cancer. It includes potent regulators of sustained proliferative signaling [KIF20A via stabilizing c-Myc (29); MCM4 as a core DNA replication helicase (30)] and agents that confer therapy resistance [G6PD (31, 32) and NDRG1 (33) by inhibiting ferroptosis; RECQL4 by mediating DNA repair and suppressing cGAS-STING-mediated immune awakening (34)]. Furthermore, it encompasses drivers of activation of invasion and metastasis (G6PD via STAT3-mediated EMT (35); NDRG1 via transcriptional regulation (36)) and deregulation of cellular energetics [G6PD as the rate-limiting enzyme of the pentose phosphate pathway (35, 37); ADH1C as a novel regulator of fatty acid degradation (38)]. Notably, the model also captures genes critical for evading immune destruction [KIF20A by inducing an immunosuppressive microenvironment and immunotherapy resistance (29); RECQL4 by inhibiting dendritic cell function (34)]. Intriguingly, ADH1C stands out as a well-validated tumor suppressor, and its loss in high-risk patients likely contributes to metabolic rewiring and loss of growth control (38). The convergence of these functionally diverse yet synergistic genes into a single predictive model, achieved through a machine learning algorithm agnostic to these pre-established functions, provides a compelling multi-hit biological rationale for the aggressive phenotype observed in high-risk patients and strongly underscores the clinical relevance of our signature.

The model’s predictive ability for treatment provides a basis for individualized medication. The low-risk group had a better response to anti-PD-1 immunotherapy, which is consistent with its immune-active microenvironment characteristics, whereas the high-risk group was more sensitive to DNA damage agents (such as the Wee1 inhibitor MK-1775) and traditional drugs (such as sorafenib). On the basis of these findings, we propose that C3 subtype/high-risk patients can adopt a synergistic strategy of cell cycle-targeted drugs combined with immunotherapy.

Thus, the ERG-centric classification system, complemented by the robust 6-gene prognostic signature, provides a clinically actionable roadmap for the personalized management of HCC patients, from prognosis assessment to therapy selection. Despite the comprehensive nature of our study, several limitations should be acknowledged. Firstly, the treatment response predictions for immunotherapy and targeted drugs are based on computational algorithms (SubMap, TIDE, oncoPredict) and public pharmacogenomic data. While these tools are widely used and validated, their predictions require confirmation in prospective clinical trials or patient-derived organoid models. Secondly, while our study establishes strong correlations and is grounded in the known biology of the signature genes, future work to experimentally validate the causal role of the entire multi-gene signature as a functional unit—using approaches such as CRISPR-based gene editing *in vivo* and *in vitro*—will be essential to fully elucidate its mechanistic role in driving aggressive HCC phenotypes and therapy resistance. Thirdly, while validated in independent cohorts, our findings warrant further validation in larger, multi-center prospective studies to ensure generalizability. Finally, integrating exosomal protein or metabolite data could further improve the accuracy of our classification and prognostic model in the future.

## Conclusion

This work establishes plasma exosomal lncRNAs as powerful biomarkers for HCC molecular subtyping, prognosis, and treatment guidance. The ERG-centric framework deciphers the lncRNA–ceRNA regulatory axis in hepatocarcinogenesis, whereas the RSF-derived risk model offers a clinically translatable tool for precision oncology. Future studies should focus on validating these findings in interventional trials and expanding multiomic integration for therapeutic discovery.

## Data Availability

The data presented in the study are deposited in the GEO repository, accession number GSE14520.
