# Supplementary material for: Plasma exosomal lncRNA-related signatures define molecular subtypes and predict survival and treatment response in hepatocellular carcinoma
Source: Front Immunol. 2025 Oct 15;16:1663943. doi: 10.3389/fimmu.2025.1663943 (PMC12568545; doi:10.3389/fimmu.2025.1663943)
Supplement: Supplementary file 1 [file Table1.docx]

| Supplementary Table S1. Primer sequences used in this study. | | |
| --- | --- | --- |
| Primer name |  | Sequence |
| ADH1C | F | 5′-CCCCAAACTTGTGGCTGACTT-3′ |
|  | R | 5′-CAGGACGGTACGGATACTCTTT-3′ |
| MCM4 | F | 5′-AGCATGGCACTCATCCACAA-3′ |
|  | R | 5′-GCACAGCTCGATAGATGCCT-3′ |
| G6PD | F | 5′-CGAGGCCGTCACCAAGAAC-3′ |
|  | R | 5′-GTAGTGGTCGATGCGGTAGA-3′ |
| KIF20A | F | 5′-AAGGGCAGAACTGGCTCATC-3′ |
|  | R | 5′-GCAAGGGCTTCAGATCAGGT-3′ |
| NDRG1 | F | 5′-CCAACAAAGACCACTCTCCTC-3′ |
|  | R | 5′-CCATGCCCTGCACGAAGTA-3′ |
| RECQL4 | F | 5′-GCGCTCTACCGGGAATACC-3′ |
|  | R | 5′-CAGCCCGATTCAGATGGGG-3′ |
| GAPDH | F | 5′-GTCTCCTCTGACTTCAACAGCG-3′ |
|  | R | 5′-ACCACCCTGTTGCTGTAGCCAA-3′ |
